# Changes in Soil Microbial Community and Its Effect on Carbon Sequestration Following Afforestation on the Loess Plateau, China

**DOI:** 10.3390/ijerph14080948

**Published:** 2017-08-22

**Authors:** Yun Xiang, Man Cheng, Yimei Huang, Shaoshan An, Frédéric Darboux

**Affiliations:** 1 College of Natural Resources and Environment, Northwest A&F University, Yangling 712100, China; xy020824@163.com (Y.X.); ymhuang1971@nwsuaf.edu.cn (Y.H.); 2 Shanxi Research Academy of Environment Sciences, Taiyuan 030000, China; 3 State Key Laboratory of Soil Erosion and Dryland Farming on the Loess Plateau, Northwest A&F University, Yangling 712100, China; chengman1988@126.com; 4 Institute of Agricultural Environment and Resource, Shanxi Academy of Agricultural Sciences, Taiyuan 030000, China; 5 French National Institute for Agricultural Research (INRA), UR 0272, UR Science du sol, Centre de recherche d’Orléans, CS 40001, Cedex 2, F-45075 Orléans, France; Frederic.Darboux@orleans.inra.fr

**Keywords:** land-use change, *C. korshinskii*, carbon fraction, soil microbial community

## Abstract

Afforestation plays an important role in soil protection and ecological restoration. The objective of this study is to understand the effect of afforestation on soil carbon and soil microbial communities in the Loess Plateau of China. We measured two chemically-separated carbon fractions (i.e., humic acid, HA, and fulvic acid, FA) and soil microbial communities within shrublands (18-year-old *Caragana korshinskii* Kom (shrubland I) and 28-year-old *Caragana korshinskii* Kom (shrubland II)) and cropland. The size and structure of the soil microbial community was measured by phospholipid fatty acid (PLFA) analysis. The analysis of C-fractions indicated that at a depth of 0–20 cm, FA-C concentration in shrubland I and shrubland II were 1.7 times that of cropland, while HA-C had similar values across all three sites. Total PLFAs, G^+^ (Gram positive) bacterial, G^−^ (Gram negative) bacterial, and actinobacterial PLFAs were highest in shrubland II, followed by shrubland I and finally cropland. Fungal PLFAs were significantly higher in shrubland II compared to the other sites. Additionally, we found a high degree of synergy between main microbial groups (apart from fungi) with FA-C. We concluded that planting *C. korshinskii* in abandoned cropland could alter the size and structure of soil microbial community, with these changes being closely related to carbon sequestration and humus formation.

## 1. Introduction

Soil organic matter (SOM) is as a critical constituent of the physical, chemical, and biological qualities of soil [1]. Restoring soil carbon is essential to enhancing soil quality, sustaining and improving food production, maintaining clean water, and reducing increases in atmospheric CO_2_ [2]. It has been suggested that alteration with different fractions of SOM are more effective in indicating changes in soil use than the total soil organic matter content. Humic substances are stable organic matter compounds which can increase moisture retention, improve soil buffering capacity, and supply plants with available micronutrients [3]. The changes in the proportion of humic substances realistically reflect carbon sequestration in soils. Based on the solubility, humic substances (HSs) can be divided into fulvic acids (FAs, alkali-extractable and acid soluble), humic acids (HAs; alkali-extractable and acid insoluble), and humin (not extractable). The changes in the proportion of chemically-separated carbon fractions in soil can provide more insights into the mechanisms of carbon sequestration.

A healthy soil comprises highly diverse biota. Soil micro-organisms also play a central role in the decomposition and composition of soil carbon [4]. Fungi and bacteria govern most of the transformations and the ensuing long-term storage of organic carbon in soils. Changes in the ratio of fungi to bacteria and the total biomass may affect the storage and fluxes of carbon (C) and nitrogen (N) in terrestrial ecosystems [5]. Six et al. noted that fungal-dominated soils had slow C turnover rates [6]. Collectively, these observations imply that the microbial community structure may change and influence C sequestration.

In the last century, the “Grain for Green” Project was conducted nationwide in order to rehabilitate and recover the degraded ecosystems in China. The conversion of cropland to shrubland on the western Loess Plateau offers opportunities to conserve soil and water, improve micro-climate, and so on. *Caragana korshinskii* is the primary afforestation species due to its capacity to limit soil erosion, its well-developed root system, its resistance to drought, and its N_2_-fixing ability [7]. Afforestation can have a significant effect on soil carbon concentration and stocks [8]. In recent years, the effects of afforestation on soil properties have been studied in China [7,8,9]. However, the microbial mechanisms of C sequestration following afforestation have not been clear. Therefore, a better understanding of the effects of soil microbial communities on soil C dynamics is fundamental for providing further insight into the microbial mechanisms of C sequestration following afforestation.

The objective of this study was to assess the impact of afforestation on the soil microbial community composition and carbon fractions (humic acid, HA, and fulvic acid, FA) in shrublands (18-year-old *C. korshinskii* and 28-year-old *C. korshinskii*) and cropland on the Loess Plateau of China. In addition, the potential links between soil microbial and carbon variations following afforestation were studied. We hypothesized that (1) there would be more soil organic carbon (SOC), FA-C, and HA-C in shrublands due to the additional input of plant residue and less human disturbance; (2) soil microbial communities would change in biomass and composition following afforestation with *C. korshinskii* planted; and (3) the different components of soil microbial community (fungi, bacteria, actinomycetes) would play different roles in controlling soil carbon sequestration.

## 2. Materials and Methods

### 2.1. Study Site and Experimental Design

**Figure 1:**
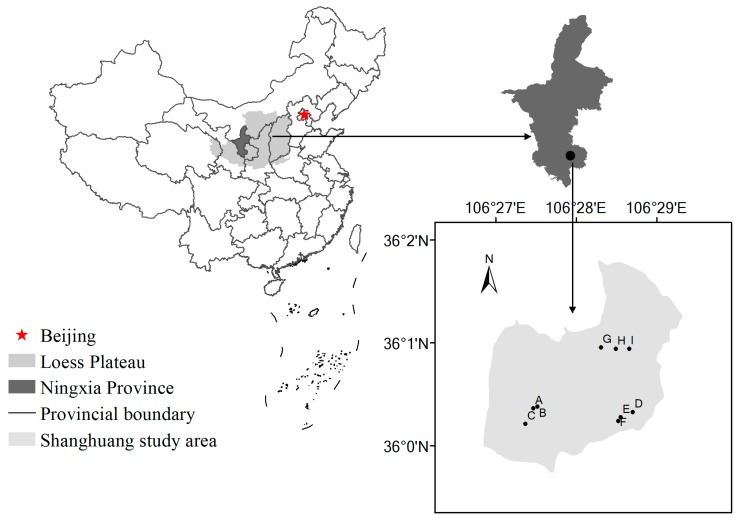
The location of the study area and sampling sites: (**A**–**C**) cropland; (**D**–**F**) 26-year-old *C. korshinskii*; in addition to (**G**–**I**) 16-year-old *C. korshinskii*.

The study was performed at Guyuan Ecological Research Station in the “hilly-gully” region of the Loess Plateau. This station is located in the Shanghuang study area (106°26′–106°30′ E, 35°59′–36°02′ N), Guyuan city, Ningxia autonomous region (Figure 1). The elevation of the study region is 1534–1822 m. The study area has a sub-arid climate. The region is characterized by a mean annual temperature of 6.9 °C, a mean annual precipitation of 419.1 mm, and an aridity index of 1.55–2.00. The soil texture is medium loam. According to the U.S. Soil Taxonomy, the studied soils were classified as Entisols [10].

In the 1980s, a pilot program of the “Grain for Green” Project was established in the Shanghuang study area. Through this program, part of the cropland was successively converted into *C. korshinskii* shrubland in 1984 and 1994. Shrubland is presently one of main vegetation types. Here, we considered two establishment durations for *C. korshinskii* shrublands: 18-year-old *C. korshinskii* shrubland (shrubland I, from 1994 to 2012) and 28-year-old *C. korshinskii* shrubland (shrubland II, from 1984 to 2012), with cropland used as a reference. Croplands are currently used by farmers for wheat production with very low fertilizer input. The locations of the sampling sites are shown in Figure 1.

### 2.2. Soil Sampling and Preparation

Soil samples were obtained from shrublands and croplands in July 2012. Three sites were chosen for each treatment. For each site, three plots of 20 m × 20 m were delineated. Five samples were collected at depths of 0–20 and 20–40 cm from each plot and mixed to form a soil sample of about 1 kg. A total of 54 soil samples were obtained from all treatments. Each sample was passed through a 2-mm sieve to remove stones, large roots, macrofauna, and so on. Part of the fresh samples was sealed in plastic bags and stored in a refrigerator at −80 °C for soil microbial community analyses, while the remaining samples were air-dried at room temperature in a laboratory for chemical and carbon fraction analysis.

### 2.3. Analysis of Soil Chemical Properties, Carbon Fractions, and Phospholipid Fatty Acid (PLFA) Profiles

The concentration (g·kg^−1^) of soil organic carbon was determined by the Walkley and Black wet oxidation method [11,12]. The total N was analyzed using the Kjeldahl method [12]. Nitrate-N and ammonium-N were measured with a continuous flow analyzer (Flowsys, SYSTEA, Anagni, Italy). The available P was measured with the NaHCO_3_ method [12]. The chemically-separated carbon fractions of soil were determined by an extraction method using a NaOH and Na_4_P_2_O_7_ mixture [11,13]. The sum of FA and HA (FA+HA) was extracted from the 2.5 g soil sample using a 50-mL mixture of 0.1 M NaOH and 0.1 M Na_4_P_2_O_7_ at 70 °C for 60 min. The dark brown alkaline solution was separated into the acid-insoluble HA and the acid-soluble FA fractions by acidifying the alkaline solution to a pH of 1.0 with 0.5 M H_2_SO_4_. The HA was washed successively with 0.05 M H_2_SO_4_ and water, before being dissolved in 0.05 M NaOH. The carbon concentrations of (FA+HA)-C and HA (HA-C) were determined through the Walkley and Black wet oxidation method, while the carbon (C) concentration of FA (FA-C) was calculated by subtracting the HA-C from the (FA+HA)-C.

Soil microbial community structure was assessed by PLFA analysis using a modified Bligh and Dyer method as described by Bossio and Scow [14]. The PLFAs were measured with a Shimadzu GCMS-QP2010 SE (Shimadzu, Tokyo, Japan). The PLFAs were analyzed using a 5% diphenyl–95% dimethyl polysiloxane stationary phase (Rtx-5MS, 0.25 mm film thickness). Methyl nonadecanoate fatty acid (19:0) was added as an internal standard to quantify peak areas. The individual PLFAs were identified using fatty acid methyl ester (FAME) standard compounds (Bacterial Acid Methyl Esters Mix; Supelco, Bellefonte, PA, USA). The total PLFAs was analyzed as an index of microbial biomass (expressed as nmol·g^−1^ soil). The primary microbial groups of bacteria, fungi, gram-positive bacteria (G^+^), and gram-negative bacteria (G^−^) were also studied to characterize the community structure. The ratio of G^+^/G^−^ bacteria and fungi/bacteria were analyzed.

### 2.4. Statistical Analysis

All data in the experiment were processed and calculated via Excel version 2003 (Microsoft, Redmond, WA, USA) and SPSS version 18.0 (IBM, Armonk, NY, USA). The effects of vegetation and soil depth on soil chemical properties, carbon fractions, and soil microbial composition were analyzed by a two-way analysis of variance (ANOVA). The Student–Newman–Keuls method (*p* < 0.05) was used to assess the differences among vegetations and soil depths. Pearson’s correlation analysis was used to study the significance of relationships between soil chemical properties, carbon fractions, and soil microbial community. Differences were considered significant if *p* < 0.05.

## 3. Results

### 3.1. Soil Chemical Properties

Soil chemical properties in shrublands and cropland are shown in Table 1. The concentrations of SOC were 1.6 and 1.1 times higher in shrubland I than in the cropland at depths of 0–20 and 20–40 cm, while it was 1.4 times higher in shrubland II than in the cropland at both depths. No significant differences in total N from shrubland I and shrubland II were observed at the depth of 0–20 cm, while the total N was highest in shrubland II at a depth of 20–40 cm, followed by shrubland I and finally cropland. NH_4_-N concentration was highest in shrubland I at both soil depths. The concentration of NO_3_-N was significantly higher in the cropland than the shrublands. For both layers, there was no difference in available P concentration between shrubland I and shrubland II. The concentration of available P was approximately 5 mg·kg^−1^ higher in cropland than in the *C. kor.* sites at a depth of 0–20 cm. At a depth of 20–40 cm, the concentration of available P was lower in cropland than in shrubland I and shrubland II. Apart from the total N in the cropland, the concentration of SOC, total NO_3_-N, and available P at a depth of 0–20 cm was consistently higher in the upper layer. The ratio of C to N varied from 6.43 to 9.48 under different vegetation types. The C/N ratio at both depths were significantly lower in shrubland II than in the cropland.

### 3.2. Soil Carbon Fraction and Its Proportion

The concentration of (HA+FA)-C had a range of 0.9–2.1 g·kg^−1^ (Table 2). At a depth of 0–20 cm, the concentrations of FA-C in shrubland I and shrubland II were significantly higher than that in the cropland (by a factor of about 1.7). At a depth of 20–40 cm, the concentrations of FA-C in the 28-year-old *C. kor.* site were significantly higher than in the other sites. HA-C in the cropland and shrubland had a range of 0.1–0.6 g·kg^−1^. There were no significant differences in HA-C concentrations among the cropland and shrublands at a depth of 0–20 cm. At a depth of 20–40 cm, HA-C concentration in increasing order was shrubland I, cropland, and shrubland II. At a depth of 0–20 cm, the proportion of FA-C in increasing order was cropland, shrubland I, and shrubland II. At a depth of 20–40 cm, the proportion of FA-C in shrubland II was significantly higher than in the other sites. In comparison, the HA-C/SOC from shrubland II and cropland was significantly higher than the shrubland I at both soil depths. The ratio of HA-C to FA-C varied from 0.17 to 0.64. At both soil layers, the ratio of HA-C to FA-C in increasing order was shrubland I, shrubland II, and cropland.

### 3.3. Soil Microbial Community Composition

A total of 36 PLFAs were detected. Twenty-eight of these PLFAs belonging to soil microbes were considered. Total PLFAs had a range of 10.9–51.0 nmol·g^−1^ soil at the two depths (Table 3). At both soil depths, total PLFAs, G^+^ bacterial, G^−^ bacterial, fungal, and actinobacterial PLFAs had the highest concentration in shrubland II, followed by shrubland I and finally cropland. Bacterial PLFAs accounted for 65–71% of the total PLFAs. At a depth of 0–20 cm, fungal PLFAs in the shrubland II site were significantly greater than that of cropland and shrubland I. For a given depth, the ratios of gram-positive/gram-negative bacteria were not statistically different between the vegetation types. These ratios were approximately 0.7 at a depth of 0–20 cm and 1.1 at a depth of 20–40 cm for all the sites. For both depths, the ratios of fungal/bacterial PLFAs were significantly lower in the shrublands than in the cropland. While these ratios were not statistically different for the shrublands between the two depths (about 1.3), it was significantly higher in the cropland at a depth of 20–40 cm than at a depth of 0–20 cm.

### 3.4. Correlation of Soil Chemical Properties, Carbon Fractions, and Microbial Community

As shown in Table 4, SOC, HS-C, and FA-C was significantly associated with total nitrogen (*p* < 0.05). There is a strong relationship between carbon fractions and soil microbial community. HS-C and FA-C were significantly correlated to the primary microbial groups, with the exception of fungi (*p* < 0.05). HA-C had a highly significant correlation with soil fungi, while SOC was observed to be strongly related to actinomyces. This indicated that the structure of the soil microbial community influenced carbon sequestration.

## 4. Discussion

### 4.1. Effect of Afforestation on Soil Carbon Sequestration

SOC is sensitive to land-use and land-cover change. Quantifying SOC is important for considerations of afforestation or reforestation [15]. In this study, we confirmed that afforestation with shrubs induced an increase in soil carbon. This finding is consistent with previous studies. Jia et al. suggested that SOC increased with the development of shrubs [7]. Additionally, An et al. found higher SOC in artificial shrublands than in croplands [8]. Throop and Archer reported that SOC increased with shrub (*Prosopis velutina*) size [16]. This increase in SOC may result from the higher carbon input and the lower rates of SOC decomposition and soil erosion associated with shrub plantation. Deng et al. reported that SOC was positively related to both litter and root biomass following afforestation [15]. Conventional tillage is typically used in the croplands on the Loess Plateau, which induces substantial carbon losses. Tillage disturbance results in accelerated SOC decomposition, before a decrease in SOC occurs [17]. In contrast, the establishment of shrubland can decrease artificial disturbance and subsequently enhance the SOC.

Furthermore, we found a higher SOC from shrubland I than shrubland II at a depth of 0–20 cm, while the results were opposite at a depth of 20–40 cm. This is related to a different mechanism of SOC sequestration for the topsoil and subsoil. This increase in SOC in topsoil may be associated with a higher carbon input and the lower rates of SOC decomposition following afforestation. The mechanisms associated with the SOC accumulation in the subsoil are different from those in the topsoil. A more extensive root system in shrubland II than shrubland I is the main factor in the higher accumulation of SOC at a depth of 20–40 cm. Qu et al. found the highest root biomass and soil organic carbon at a depth of 20–40 cm in 26-year-old *C. korshinskii* shrubland compared to 10-year-old, 40-year-old, and 50-year-old *C. korshinskii* shrubland [18].

Fulvic acid (FA) and humic acid (HA) form an important part of the organic matter in soil. Afforestation had a positive influence on FA-C and little influence on HA-C. This result is consistent with Guimarães et al., who reported that the HA fraction did not differ significantly with different soil uses [19]. However, this is not fully consistent with Reddy et al., who suggested that both HA-C and FA-C were higher in forest soils compared to agricultural soils [20]. Furthermore, we found an increase in the proportion of FA-C with C sequestration. This phenomenon implied that FA-C increased when the total organic carbon accumulated in soil after *C. korshinskii* plantation. The ratio of humic and fulvic acids (HA-C/FA-C) reflects the potential mobility of soil organic carbon. For shrublands and cropland, FA-C predominated with a HA-C/FA-C < 1. This indicated frequent inputs of fresh organic residues [19]. Moreover, the highest HA-C/FA-C was observed in cropland, followed by shrubland II, while the lowest was in the shrubland I. This indicated a substantial increase in fulvic acids following afforestation, especially in the early stage. According to the polyphenol theory, the structure of HA is believed to be an aromatic ring of the di- or trihydroxy phenyl type, bridged together by -O-, -CH_2_- and other groups [21]. In comparison, FA has a lower molecular weight and higher mobility than HA. The formation of FA occurs prior to the formation of HA. Above all, we conclude that afforestation enhanced FA-C in soil with frequent inputs of fresh organic residues.

### 4.2. Effect of Afforestation on Soil Microbial Community

Our results clearly demonstrated that afforestation had significant effects on the size and structure of soil microbial communities. Compared to the cropland, the microbial biomass that was marked by total PLFAs significantly increased after afforestation and increased with time after planting. This finding is consistent with previous studies. For instance, Cao et al. observed that shrub growth improved soil microbial biomass [22], while Jia et al. found that *C. korshinskii* that was planted on abandoned land gradually had a positive effect on the soil microbial biomass [7]. This higher bacterial biomass found in the shrubland soils is consistent with Hortal et al., who reported that the bacterial biomass increased with shrub size [23]. The fungal biomass was only observed significantly higher in shrubland II than the cropland in 0–20 cm layer. This finding is not precisely consistent with previous results. Hortal et al. reported that the fungal biomass increased with shrub size [23], while Bossio et al. found that afforestation improved fungal biomass and richness in addition to altering the microbial community structure (measured by the PLFA analysis) [24]. The microbial biomass and bacterial biomass strongly depend on plant productivity and carbon input. The greater amounts of soil microbial biomass suggested that more resources are available in shrubland soils than in cropland soils [25]. The higher concentration of SOC in shrubland soils may explain this phenomenon. According to the fungal biomass, we consider it to be related to the quality of plant residue and soil organic matter. Changes in the fungal biomass following afforestation were attributed to an increase in soil organic matter after the removal of soil disturbance.

Changes in the composition of soil microbial community (e.g., the ratios of fungi/bacteria and gram-positive bacteria/gram-negative bacteria) positively correlate with soil nutritional stress or negatively with resource availability. We found similar G^+^/G^−^ bacterial distributions in shrublands and cropland (0.6 at a depth of 0–20 cm and 1.0 at a depth of 20–40 cm), and lower fungi/bacteria in the shrubland soils than in the agricultural soils. The results are inconsistent with Macdonald et al., who reported increasing fungi/bacteria ratios after afforestation [26]. However, these results are consistent with Bailey [5], who showed a lower fungi/bacteria ratio in forest soils compared to agriculture field soils. Furthermore, Hortal et al. showed that the fungi/bacteria ratio decreased as the plant grows (*Retama sphaerocarpa Boiss*, a leguminous shrub) [23]. *C. korshinskii* is a deciduous leguminous shrub that has a strong N_2_-fixing ability. These results suggest that afforestation with planting leguminous shrub could significantly cause a decrease in fungi/bacteria. The *C. korshinskii* shrubland soils have a large number nitrogen-fixing microorganisms, such as rhizobacter, ammonifiers and nitrobacteria [27]. These microorganisms might contribute to the higher gram-negative bacterial biomass in shrublands soils [27], which resulted in a lower fungi/bacteria ratio.

### 4.3. Changes in Soil Microbial Community Control Soil Carbon Sequestration

Soil microbial community and carbon cycling are complex processes following afforestation. Our results showed a strong relationship between SOC, FA-C, and soil microbial community. Soil organic carbon was closely related to actinomycetes biomass. Nsabimana et al. suggested that land use had substantial effects on the size and diversity of the soil microbial community, with these changes possibly being broadly related to changes in the SOC [28]. We also found that FA-C was correlated with these main microbial groups except for fungi, while HA-C was related to fungal biomass. Jastrow et al. suggested that manipulations enhancing the fungal community and (bio) chemistry associated with the humidification process had the potential to increase the sequestration of C in soils [29]. Therefore, we further concluded that planting *C. korshinskii* resulted in significant changes in the bacterial and fungal biomass. These changes were closely related to carbon sequestration and humus formation.

## 5. Conclusions

Changes in carbon fractions and soil microbial community were assessed in the response to afforestation from abandoned cropland on the Loess Plateau of China. Our results indicated that afforestation caused increases in the G^+^, G^−^, total bacterial, and actinomycetes biomass. Afforestation positively influenced SOC and FA-C. Additionally, we found that the changes in bacteria and actinomycetes were closely related to FA-C, while fungal biomass was strongly correlated with HA-C. These responses indicated that planting *C. korshinskii* in abandoned cropland could alter the size and structure of soil microbial community. Furthermore, these changes were closely related to carbon sequestration and the humus formation. The establishment of *C. korshinskii* shrubland in this area promoted soil organic carbon sequestration and soil ecological environment.

## Figures and Tables

**Table 1:** Soil chemical and physical properties (mean ± standard error, *n* = 9).

Vegetation Type	Depth (cm)	SOC	Total·N	NH_4_-N	NO_3_-N	Available *p*	C/N	Bulk Density
		g·kg^−1^	g·kg^−1^	mg·kg^−1^	mg·kg^−1^	mg·kg^−1^		g·cm^−3^
Cropland	0–20	7.66 ± 0.10Ca	0.89 ± 0.19Ba	3.75 ± 0.50Ba	13.10 ± 0.05Aa	9.37 ± 0.54Aa	8.61 ± 0.29Aa	1.30
	20–40	6.35 ± 0.28Cb	0.67 ± 0.02Ca	3.69 ± 0.26Ca	8.08 ± 0.38Ab	2.55 ± 0.13Bb	9.48 ± 0.57Aa	1.51
shrubland I	0–20	12.39 ± 0.15Aa	1.45 ± 0.06Aa	6.02 ± 0.12Aa	7.46 ± 0.18Ba	4.89 ± 0.21Ba	8.55 ± 0.27Aa	1.04
	20–40	7.43 ± 0.18 Bb	0.92 ± 0.07Bb	5.71 ± 0.03Aa	6.41 ± 0.20Bb	3.57 ± 0.20Ab	8.14 ± 0.84ABa	1.14
shrubland II	0–20	10.38 ± 0.37Ba	1.59 ± 0.07Aa	5.07 ± 0.23Ba	7.67 ± 0.20Ba	4.24 ± 0.13Ba	6.52 ± 0.37Ba	1.17
	20–40	8.80 ± 0.02Ab	1.37 ± 0.09Ab	4.37 ± 0.06Ba	5.96 ± 0.19Bb	3.31 ± 0.19Ab	6.43 ± 0.25Ba	1.30

Different lowercase letters indicate significant differences at *p* < 0.05 at soil depths for each vegetation type; different capital letters indicate significant differences at *p* < 0.05 among different vegetation types at a same depth. SOC = soil organic carbon.

**Table 2:** Soil organic carbon fraction concentrations and their proportion to total SOC.

Vegetation Type	Depth (cm)	FA-C (Fulvic Acid)		HA-C (Humic Acid)		HA-C/FA-C
		Concentration (g·kg^−1^)	Proportion to Total SOC (%)	Concentration (g·kg^−1^)	Proportion to Total SOC (%)	
Cropland	0–20	0.76 ± 0.13Ba	9.9C	0.48 ± 0.01Aa	6.3A	0.63 ± 0.09Aa
	20–40	0.63 ± 0.13Ba	10.0B	0.28 ± 0.01Bb	4.4A	0.45 ± 0.10Aa
shrubland I	0–20	1.64 ± 0.06Aa	13.2B	0.44 ± 0.01Aa	3.5B	0.27 ± 0.01Ca
	20–40	0.78 ± 0.02Bb	10.4B	0.13 ± 0.01Cb	1.7B	0.17 ± 0.02Ca
shrubland II	0–20	1.61 ± 0.05Aa	15.6A	0.54 ± 0.01Aa	5.2A	0.34 ± 0.08Ba
	20–40	1.17 ± 0.03Ab	13.3A	0.41 ± 0.03Ab	4.7A	0.35 ± 0.01Ba

Different lowercase letters indicate significant differences at *p* < 0.05 within soil depths for each vegetation type; different capital letters indicate significant differences at *p* < 0.05 among different vegetation types at the same depth.

**Table 3:** The concentrations of total phospholipid fatty acids (PLFAs), Gram-positive (G^+^) bacterial, Gram-negative (G^−^) bacterial, total bacterial, fungal, actinomycetic PLFAs (nmol·g^−1^·soil, mean ± standard error, *n* = 9) and the ratio of G^+^:G^−^ bacterial PLFAs and fungal:bacterial PLFAs in the soils of cropland and shrublands.

Vegetation Type	Depth (cm)	Total PLFAs	G^+^ Bacterial PLFAs ^a^	G^−^ Bacterial PLFAs ^b^	Bacterial PLFAs ^c^	Fungal PLFA ^d^	Actinomycetic PLFAs ^e^	G^+^:G^−^ Bacterial PLFAs	Fungal:Bacterial PLFAs
Cropland	0–20	29.17 ± 0.99Ca	4.79 ± 0.34Ca	7.42 ± 0.21Ca	20.30 ± 0.89Ca	3.83 ± 0.12Ba	1.47 ± 0.03Ca	0.64 ± 0.03Ab	2.61 ± 0.14Ab
	20–40	10.89 ± 0.48Cb	1.53 ± 0.16Cb	1.40 ± 0.09Cb	7.12 ± 0.23Cb	2.20 ± 0.11Ab	0.42 ± 0.01Cb	1.09 ± 0.05Aa	5.25 ± 0.16Aa
shrubland I	0–20	35.98 ± 0.38Ba	6.55 ± 0.34Ba	9.23 ± 0.35Ba	24.95 ± 0.49Ba	3.50 ± 0.35Ba	2.50 ± 0.05Ba	0.71 ± 0.01Ab	1.41 ± 0.17Ba
	20–40	19.04 ± 0.50Bb	3.93 ± 0.03Bb	3.89 ± 0.25Bb	13.31 ± 0.21Bb	1.78 ± 0.20Bb	1.44 ± 0.08Bb	1.01 ± 0.07Aa	1.24 ± 0.21Ba
shrubland II	0–20	51.02 ± 1.57Aa	9.33 ± 0.59Aa	14.18 ± 0.06Aa	36.14 ± 0.99Aa	4.54 ± 0.11Aa	3.19 ± 0.34Aa	0.66 ± 0.04Ab	1.43 ± 0.12Ba
	20–40	28.62 ± 1.03Ab	6.48 ± 0.40Ab	5.77 ± 0.36Ab	19.86 ± 0.78Ab	2.53 ± 0.19Ab	2.22 ± 0.19Aa	1.12 ± 0.02Aa	1.15 ± 0.18Ba

Different lowercase letters indicate significant differences at *p* < 0.05 between soil depths for each vegetation type; different capital letters indicate significant differences at *p* < 0.05 among different vegetation types at a same depth. ^a^ a14:0, i14:0, i15:0, a16:0, i16:0, a17:0, i17:0; ^b^ 16:1ω5c, 16:1ω7, 16:1ω9, cy17:0, 18:1ω7, cy19:0, 16:1ω7t, 17:1ω8c; ^c^ 14:0, 15:0, 16:0, 17:0, 18:0, 20:0 and G^+^, G^−^ bacterial PLFAs; ^d^ 18:2ω6,9, 18:1ω9c, 18:1ω9t; ^e^ 10Me 16:0, 10Me 17:0, 10Me 18:0.

**Table 4:** Correlation coefficient (*r*) matrix between soil microbial groups and indicators of soil chemistry and carbon fractions.

	SOC	FA-C	HA-C	HA-C/FA-C
**Soil Chemical and Physical Properties**				
TN	0.869 *	0.957 **	0.622	–0.376
C/N	–0.358	–0.554	–0.414	0.270
NO_3_-N	–0.222	–0.325	0.396	0.866 *
NH_4_-N	0.682	0.613	–0.177	–0.853 *
Available P	0.054	–0.082	0.470	0.671
Bulk density	–0.758	–0.855 *	–0.610	0.309
**Soil Microbial Community**				
Total PLFAs	0.766	0.861 *	0.790	–0.074
G^+^ Bacterial	0.763	0.875 *	0.711	–0.206
G^−^ Bacterial	0.747	0.831 *	0.782	–0.041
Bacterial	0.758	0.855 *	0.783	–0.078
Fungi	0.587	0.636	0.916 *	0.362
Actinomycetes	0.832 *	0.921 **	0.643	–0.321
G^+^/G^−^	–0.552	–0.490	–0.679	–0.292
F/B	–0.604	–0.607	–0.183	0.522

* and ** indicate the significance at *p* < 0.05 and *p* < 0.01, respectively. Definitions: TN = Total nitrogen; HA-C = humic acid; FA-C = ulvic acid; G^+^ refers to gram-negative bacteria; and G^−^ refers to gram-positive bacteria.
